# Supplementary material for: Accelerating Ligand Discovery for Insect Odorant Receptors
Source: Int J Biol Sci. 2025 Feb 18;21(5):2101–17. doi: 10.7150/ijbs.105648 (PMC11900807; doi:10.7150/ijbs.105648)
Supplement: Supplementary file 1 — Supplementary figures and tables 2-9. [file ijbsv21p2101s1.pdf]

Supplementary Material

Accelerating Ligand Discovery for Insect Odorant Receptors

Arthur Comte[1,2], Maxence Lalis[1], Ludvine Brajon[2], Riccardo Moracci[2], Nicolas Montagné[2,3], Jérémie Topin[1], Emmanuelle Jacquin Joly[2]*, Sébastien Fiorucci[1]*

* corresponding authors: sebastien.fiorucci@univ-cotedazur.fr, emmanuelle.joly@inrae.fr

[1] Université Côte d'Azur, Institut de Chimie de Nice (ICN) UMR 7272, CNRS, 06008 Nice, France

[2] INRAE, Sorbonne Université, CNRS, IRD, Université Paris Cité, Université Paris Est Créteil Val de Marne, Institut d'Ecologie et des Sciences de l'Environnement de Paris (iEES-Paris), Versailles cedex 78026, France

[3] Institut universitaire de France (IUF)

Contents

Table S1: List of molecules used to assess the docking protocol, compounds tested during electrophysiological assays and known ligands of SlitOR25 and SlitOR31. New ligands of SlitOR25 and SlitOR31 discovered with this present study are highlighted in grey.

Figure S1: The AlphaFold2 and AlphaFold3 models of insect ORs suggest high structural quality, particularly in the binding region, as highlighted by their pLDDT scores.

Figure S2: The binding sites of the 17 SlitORs models examined in this study, generated by either AlphaFold 2 or 3, exhibit a perfect match with the one identified in the experimental structure of MhraOR5.

Table S2: LE stands out as the top-performing scoring function for analyzing the odorant docking poses on non-pheromonal odorant receptors of *Spodoptera littoralis*, including SlitOR25 and SlitOR31.

Figure S3: The predicted chemical space of SlitOR25 and SlitOR31 is divided into 68 and 70 molecular clusters, respectively.

Table S3: Performance of the *in silico* prediction for SlitOR25 and SlitOR31.

Table S4: Predicted vapor pressure of compounds tested during single-sensillum recordings.

Table S5: Summary of the residues in the binding pockets of SlitOR25 and SlitOR31 that were aligned with amino acids in the binding pockets of DmelOrco[2], MhraOR5[3], ApisOR5[5] and AaegOR10[6], where mutations have been shown to significantly impact ligand-receptor interactions.

Table S6: Comparison of AlphaFold predictions and template-based models (TBM) for predicting insect odorant receptor structures.

Table S7: SlitOR25 and SlitOR31 exhibit low sequence identity compared to the currently available insect odorant receptor cryo-EM structures.

Figure S4: Effect of molecular weight and chemical family classification on the performance of Vinardo and LE scoring functions.

Figure S5: Effect of the p-value threshold on the performance of the *in silico* prediction.

Figure S6: Correlation between pocket descriptors and OR tuning.

Table S8: Correlation matrix.

Figure S7: The recognition of eugenol by the broad receptor MhraOR5 involves fewer polar interactions compared to its recognition by the specific receptor SlitOR31.

Figure S8: Linear regression models for the prediction of OR broadness based on the description of the binding pocket.

Table S9: Performance of the linear regression models.

Table S1: List of molecules used to assess the docking protocol. compounds tested during electrophysiological assays and known ligands of SlitOR25 and SlitOR31. New ligands of SlitOR25 and SlitOR31 discovered with this present study are highlighted in grey.

Table S1 is provided in the *Table_S1.xls* file

A

		AlphaFold2					AlphaFold3				
		Model 1	Model 2	Model 3	Model 4	Model 5	Model 1	Model 2	Model 3	Model 4	Model 5
AaegOR10	RMSD to cryo-EM	1.338 Å	**1.304 Å**	1.684 Å	1.691 Å	1.737 Å	**1.459 Å**	1.519 Å	1.490 Å	1.466 Å	1.744 Å
	pLDDT score	86.563	**90.651**	88.972	89.612	90.324	**86.838**	86.828	86.568	86.592	85.906
AgamOR28	RMSD to cryo-EM	2.574 Å	1.923 Å	2.195 Å	**1.834 Å**	1.983 Å	**1.892 Å**	2.018 Å	1.984 Å	2.236 Å	1.948 Å
	pLDDT score	84.560	85.640	85.419	**85.934**	83.916	**79.486**	79.251	79.201	79.237	79.066
ApisOR5	RMSD to cryo-EM	2.215 Å	2.102 Å	2.354 Å	**2.034 Å**	2.225 Å	1.614 Å	1.789 Å	1.542 Å	1.657 Å	**1.438 Å**
	pLDDT score	85.494	85.811	86.378	**86.637**	79.879	79.344	79.505	79.309	78.803	**83.487**
MhraOR5	RMSD to cryo-EM	2.230 Å	**1.958 Å**	2.404 Å	2.091 Å	2.072 Å	**2.084 Å**	2.314 Å	2.060 Å	1.904 Å	2.044 Å
	pLDDT score	75.386	**76.227**	72.907	72.278	72.164	**71.338**	71.252	71.050	71.112	71.142
AbakOrco	RMSD to cryo-EM	3.826 Å	1.458 Å	**1.174 Å**	1.446 Å	4.186 Å	4.152 Å	**1.088 Å**	2.888 Å	3.092 Å	2.893 Å
	pLDDT score	80.108	77.702	**80.232**	78.058	78.254	76.480	**77.066**	76.600	76.201	76.688

B

		AlphaFold2					AlphaFold3				
		Model 1	Model 2	Model 3	Model 4	Model 5	Model 1	Model 2	Model 3	Model 4	Model 5
SlitOR25	All sequence	87.912	86.189	**88.386**	88.019	83.982	85.529	**85.556**	85.285	85.443	85.406
	Binding region	90.757	89.349	**89.867**	91.046	88.376	85.532	**85.490**	85.131	85.460	85.348
SlitOR31	All sequence	88.400	88.622	88.404	**88.701**	85.316	83.090	83.077	83.100	**83.203**	82.933
	Binding region	90.916	90.611	88.528	**90.105**	86.197	84.624	84.440	84.520	**84.607**	84.640

C

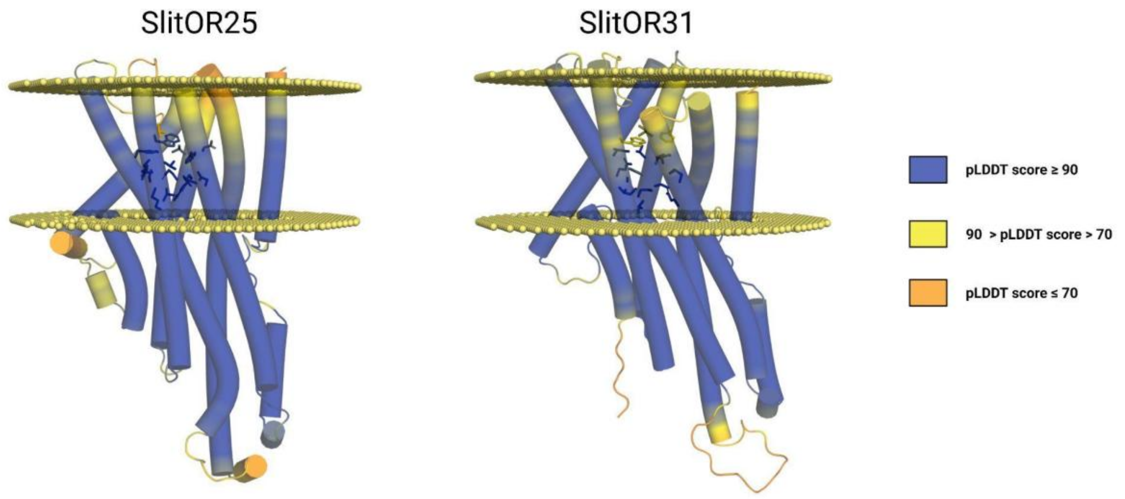

Figure S1: The AlphaFold2 and AlphaFold3 models of insect ORs suggest high structural quality, particularly in the binding region, as highlighted by their pLDDT scores. (**A**) Evaluation of the AlphaFold2 and AlphaFold3 pLDDT scoring function. Five models were generated using AlphaFold2 and AlphaFold3 for all available cryo-EM structures of insect odorant receptors[2,3,5,6]. The RMSD between each model and its experimental structure was calculated and associated with the pLDDT score for the entire protein sequence. The model with the smallest RMSD among the five generated for each receptor is highlighted in bold and blue, while the model with the highest pLDDT score is highlighted in bold and red. (**B**) pLDDT scores of the models generated using AlphaFold2 and AlphaFold3 for SlitOR25 and SlitOR31. The pLDDT scores were calculated for both the entire sequence and the binding region of the receptors. Models with the highest pLDDT score for the entire sequence were selected and are highlighted in red boxes. (**C**) AlphaFold2 models of SlitOR25 and SlitOR31 colored according to pLDDT scores: blue (≥90), yellow (70–90), and orange (<70). The side chains of residues in the binding region are shown as sticks. The receptors orientation in the membrane was determined using OPM server[65]. This figure was generated using the molecular visualization software PyMol[49].

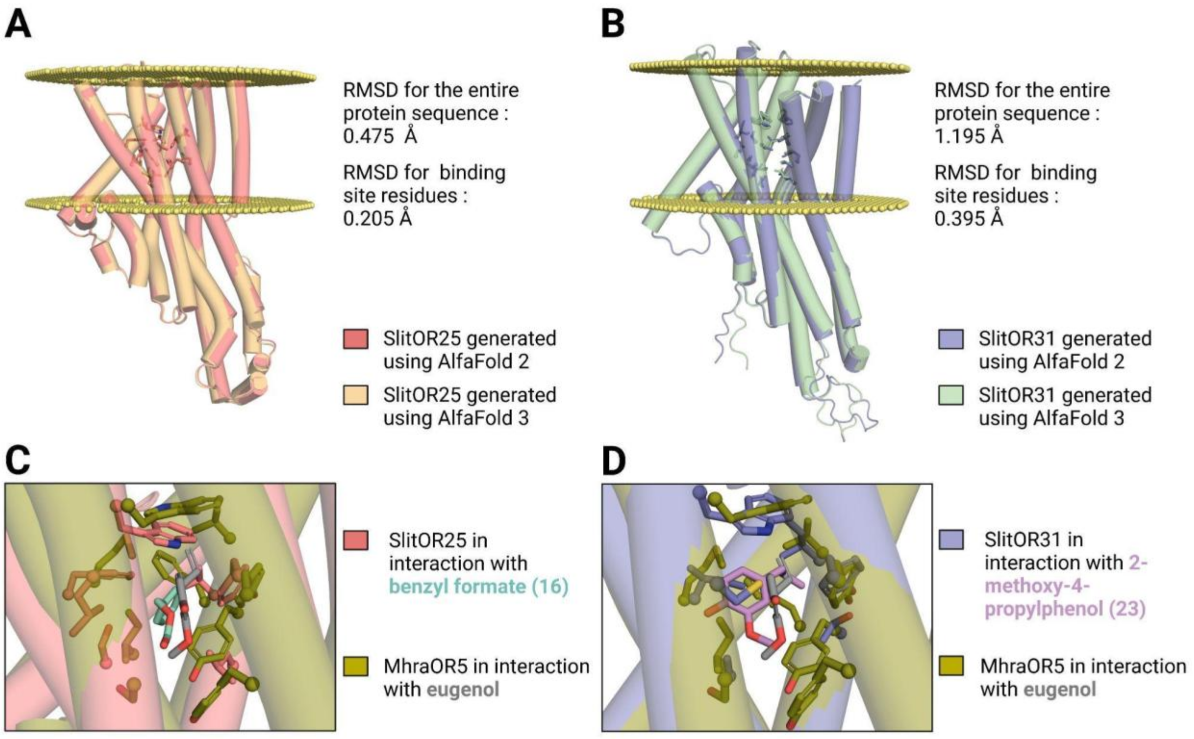

Figure S2: The binding sites of the 17 SlitORs models examined in this study, generated by either AlphaFold 2 or 3, exhibit a perfect match with the one identified in the experimental structure of MhraOR5. Comparison of the structures generated by AlphaFold2 and AlphaFold3 for (**A**) SlitOR25 and (**B**) SlitOR31. (**C**) Superposition of the binding site from AlphaFold2 models of SlitOR25 and (**D**) SlitOR31 with the experimental structure of the MhraOR5, which binds eugenol. SlitOR25 is shown interacting with benzyl formate (16), while SlitOR31 interacts with 2-methoxy-4-propylphenol (23). The receptors orientation in the membrane was determined using OPM server[65]. This figure was generated using the molecular visualization software PyMol[49].

Table S2: LE stands out as the top-performing scoring function for analyzing the odorant docking poses on non-pheromonal odorant receptors of *Spodoptera littoralis*, including SlitOR25 and SlitOR31. The comparison table summarizes the maximum enrichment factor values (Max_EF) obtained for the indicated threshold using Vinardo, LESA, LEln, and LE scoring functions based on optimal poses of 51 molecules across 17 receptors from de Fouchier et al., 2017[20]. The LESA, LEln, and LE functions are weighted versions of the Vinardo function as described in methodology. The gradient color scheme highlights AUC values, with green indicating higher performance and red lower ones.

	Max_EF_Vinardo	Threshold_Vinardo (%)	Max_EF_LEln	Threshold_LEln (%)	Max_EF_LESA	Threshold_LESA (%)	Max_EF_LE	Threshold_LE (%)
**Pheromone Receptors**								
SlitOR6	5.1	20	3.92	25	1.31	76	1.11	90
SlitOR13	12.75	4	12.75	8	2.68	37	1.46	69
**Median**	**8.93**	**12**	**8.34**	**17**	**2.00**	**57**	**1.29**	**80**
**Non-pheromonal Receptors**								
SlitOR3	2.32	4	2.32	8	1.45	63	1.55	53
SlitOR4	1.16	86	3.4	6	5.10	4	10.20	2
SlitOR7	1.06	94	1.04	96	1.38	73	2.27	29
SlitOR14	1.11	90	1.34	75	4.25	12	12.75	2
SlitOR17	1.89	53	1.82	55	2.55	16	6.80	6
SlitOR19	1.09	92	1.21	82	2.12	31	6.38	8
SlitOR24	1.16	86	1.76	57	5.67	2	5.67	2
SlitOR25	1.15	80	1.57	59	2.24	14	3.92	2
SlitOR27	1.00	100	1.00	100	1.14	63	2.43	6
SlitOR28	1.06	94	1.11	90	1.28	78	2.43	14
SlitOR29	1.24	80	1.11	90	2.43	59	2.02	35
SlitOR31	1.34	75	2.04	49	5.10	10	8.50	6
SlitOR32	1.09	92	1.19	84	1.42	47	5.67	2
SlitOR35	1.75	24	2.14	27	3.00	2	3.00	2
SlitOR36	2.83	18	4.25	23	4.63	22	8.50	2
**Median**	**1.16**	**86**	**1.57**	**59**	**2.43**	**22**	**5.67**	**6**

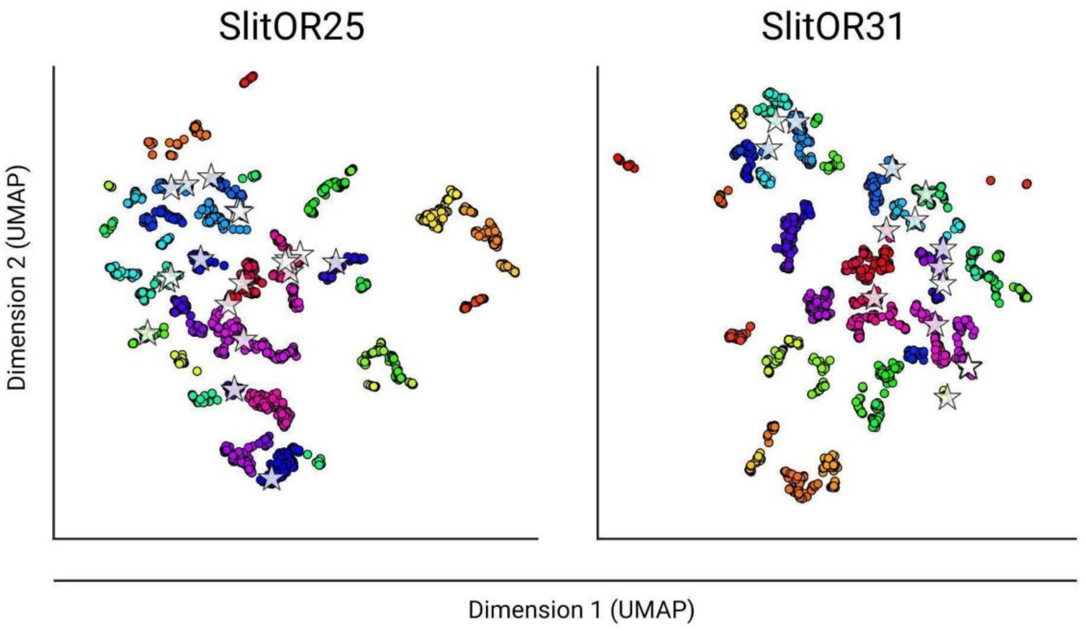

Figure S3: The predicted chemical space of SlitOR25 and SlitOR31 is divided into 68 and 70 molecular clusters, respectively. UMAP representation of the chemical space based on Morgan2 fingerprints of potential binders. The chemical space is divided into distinct clusters, each representing a group of potential binders, with each cluster uniquely colored. Tested potential binders are marked by white stars.

Table S3: Performance of the *in silico* prediction for SlitOR25 and SlitOR31. P: Positives, N: Negatives, TP: True Positives, TN: True Negatives, FP: False Positives, FN: False Negatives.

	Equations	SlitOR25	SlitOR31
**Sensitivity**	TPR = TP / (TP + FN)	1.00	1.00
**Specificity**	SPC = TN / (FP + TN)	0.32	0.33
**Precision**	PPV = TP / (TP + FP)	0.11	0.06
**Negative Predictive Value**	NPV = TN / (TN + FN)	1.00	1.00
**False Positive Rate**	FPR = FP / (FP + TN)	0.68	0.67
**False Discovery Rate**	FDR = FP / (FP + TP)	0.89	0.94
**False Negative Rate**	FNR = FN / (FN + TP)	0.00	0.00
**Accuracy**	ACC = (TP + TN) / (P + N)	0.37	0.36
**F1 Score**	F1 = 2TP / (2TP + FP + FN)	0.19	0.11
**Matthews Correlation Coefficient**	MCC = TP*TN - FP*FN / sqrt((TP+FP)*(TP+FN)*(TN+FP)*(TN+FN))	0.18	0.14

Table S4: Predicted vapor pressure of compounds tested during single-sensillum recordings. Vapor pressure (VP; mmHg) values were calculated using EPI Suite 4.11. Predicted binders for SlitOR25 and SlitOR31 are highlighted in green, potential non-binders in orange, and suspected decoys in red. The main known binders and newly identified ones for SlitOR25 and SlitOR31 are shaded in grey.

Compound	Predicted activity	Experimental activity	VP (mmHg)
acetophenone		SlitOR25 major known binder	3.26E-01
1	Predicted binders for SlitOR25	non-binder	1.13E-01
2		non-binder	1.48E-04
3		non-binder	2.75E-03
4		non-binder	2.81E-01
5		non-binder	3.99E-01
6		non-binder	4.18E-03
7		non-binder	2.04E-01
8		non-binder	8.55E-01
9		non-binder	2.10E-04
10		binder	3.22E-07
11		non-binder	3.46E-05
12		non-binder	3.68E-03
13		non-binder	9.56E-04
14		non-binder	8.40E-04
15		non-binder	5.12E-04
16		binder	3.10E-01
17		non-binder	3.56E-02
18		non-binder	4.18E-01
19		non-binder	2.40E-01
eugenol		SlitOR31 major known binder	8.54E-03
5	Predicted binders for SlitOR31	non-binder	3.99E-01
20		non-binder	7.56E-05
21		non-binder	1.66E-04
22		non-binder	7.91E-02
23		binder	2.13E-03
24		non-binder	7.59E-06
25		non-binder	1.31E-03
26		non-binder	1.21E-04
27		non-binder	2.72E-04
28		non-binder	6.15E-08
29		non-binder	3.29E-05
30		non-binder	7.49E-04
31		non-binder	5.11E-01
32		non-binder	2.25E-04
33		non-binder	5.95E-05
34		non-binder	2.48E-02
35		non-binder	3.65E-02
36	Potential non-binders for SlitOR25 and SlitOR31	non-binder	1.11E-12
37		non-binder	2.33E-14
38		non-binder	4.24E-06
39		non-binder	7.10E-09
40		non-binder	4.37E-11
41	Suspected decoys for SlitOR25 and SlitOR31	non-binder	3.74E+00
42		non-binder	2.17E+01
43		non-binder	1.86E+02

Table S5: Summary of the residues in the binding pockets of SlitOR25 and SlitOR31 that were aligned with amino acids in the binding pockets of DmelOrco[66], MhraOR5[3], ApisOR5[5] and AaegOR10[6], where mutations have been shown to significantly impact ligand-receptor interactions. Colors indicate the impact of mutations on ligand recognition: grey highlights mutations that abolished the ligand recognition, blue denotes mutations that decreased receptor sensitivity, and orange indicates mutations that increased receptor sensitivity.

SlitOR25	SlitOR31	MhraOR5	DmelOrco	ApisOR5	AaegOR10
	F78				
V88	I79	V88D		I63A	L67A
L91	T82	Y91A	F83A		
Q92		F92A	F84A		
	I141	S151A	S146I		
T153	C144	G154A			
L154	G145				S133A
W157	W148	W158A		F115A	F136A
F197	I189	M209A<br>M209L	V206W	V164A	
I200					
S201	A193	I213A		H168A	
V204					
M205					
F322	Y313	Y380A			
Y325		Y383A			

Table S6: Comparison of AlphaFold predictions and template-based models (TBM) for predicting insect odorant receptor structures. RMSD values between the available cryo-EM structures of insect odorant receptors and their respective AlphaFold and TBM models are presented. TBM models were generated for AaegOR10, AgamOR28, ApisOR5, MhraOR5, and AbakOrco using all available cryo-EM structures of insect receptors[2,3,5,6] (Protein Data Bank accessions 8V02, 8V3D, 8Z9A, 7LID, and 6C70) as templates, excluding the target itself. The color scale for RMSD values ranges from green to red, with green indicating models closely matching the cryo-EM structures and red indicating greater deviations.

	AlphaFold2	AlphaFold3	TBM_template_AaegOR10	TBM_template_AgamOR28	TBM_template_ApisOR5	TBM_template_MhraOR5	TBM_template_AbakOrco
**AaegOR10**	1.304 Å	1.459 Å		2.775 Å	4.314 Å	3.579 Å	1.328 Å
**AgamOR28**	1.834 Å	1.892 Å	3.024 Å		4.174 Å	3.555 Å	1.516 Å
**ApisOR5**	2.034 Å	1.438 Å	3.437 Å	4.165 Å		3.456 Å	1.809 Å
**MhraOR5**	1.958 Å	2.084 Å	5.489 Å	4.692 Å	4.507 Å		4.171 Å
**Abakorco**	1.174 Å	1.088 Å	4.070 Å	5.077 Å	5.561 Å	4.086 Å	

Table S7: SlitOR25 and SlitOR31 exhibit low sequence identity compared to the currently available insect odorant receptor cryo-EM structures. Pairwise sequence identity of AaegOR10, AgamOR28, ApisOR5, MhraOR5, SlitOR25, SlitOR31 and AbakOrco, using the Needleman-Wunsch algorithm.

	AaegOR10	AgamOR28	ApisOR5	MhraOR5	SlitOR25	SlitOR31	Abakorco
**AaegOR10**							
**AgamOR28**	20.0						
**ApisOR5**	18.2	16.9					
**MhraOR5**	17.6	17.0	16.5				
**SlitOR25**	16.4	18.1	21.2	16.5			
**SlitOR31**	20.5	17.1	17.4	15.8	18.8		
**Abakorco**	15.1	13.9	12.9	20.7	17.3	19.9	

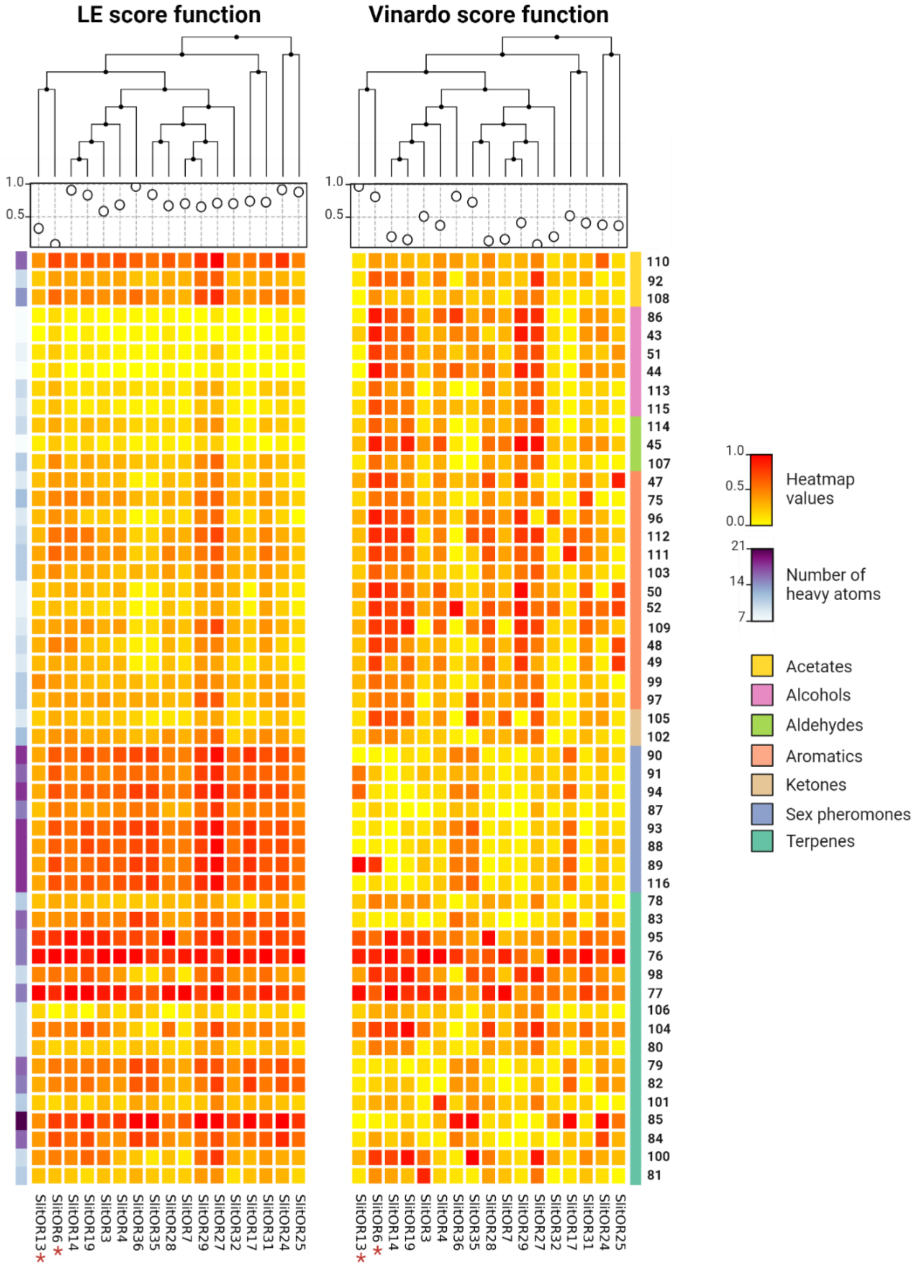

Figure S4: Effect of molecular weight and chemical family classification on the performance of Vinardo and LE scoring functions. The two heatmaps depict the deviation between normalized experimental SSR values[20] and normalized docking values: the redder the color, the larger the deviation, indicating a greater difference between docking predictions and experimental values. Conversely, as the color tends towards yellow, docking values better align with experimental ones. The heatmap on the left was generated using the LE scoring function, while the one on the right was constructed using the unweighted Vinardo function. On the left side of the heatmaps, a blue gradient represents the number of heavy atoms for each molecule in rows: darker shades indicate higher atom counts. Above the heatmaps, a dot plot displays AUC values for all receptors in columns, and a dendrogram illustrates the clustering of these receptors. On the right of the heatmaps, the color code indicate the chemical families associated with each molecule: acetates in yellow, alcohols in pink, aldehydes in light green, aromatics in red, ketones in brown, sexual pheromones in blue, and terpenes in dark green. Molecules are labeled as in Supplementary Table 1. A red star denotes pheromone receptors.

A

	LE (p-value < 0.001)	LE (0.001< p-value < 0.01)	LE (0.01 < p-value < 0.05)
**Pheromone Receptors**			
SlitOR6	0.10	0.10	0.10
SlitOR13	0.34	0.33	0.33
**Median**	**0.22**	**0.22**	**0.22**
**Non-pheromonal Receptors**			
SlitOR3	0.59	0.67	0.80
SlitOR4	0.69	0.69	0.69
SlitOR7	0.71	0.65	0.75
SlitOR14	0.91	0.85	0.84
SlitOR17	0.75	0.84	0.84
SlitOR19	0.83	0.86	0.84
SlitOR24	0.92	0.92	0.85
SlitOR25	0.87	0.87	0.87
SlitOR27	0.72	0.77	0.81
SlitOR28	0.68	0.79	0.77
SlitOR29	0.66	0.67	0.57
SlitOR31	0.72	0.72	0.82
SlitOR32	0.71	0.74	0.74
SlitOR35	0.85	0.90	0.89
SlitOR36	0.97	0.98	0.93
**Median**	**0.72**	**0.79**	**0.82**

B

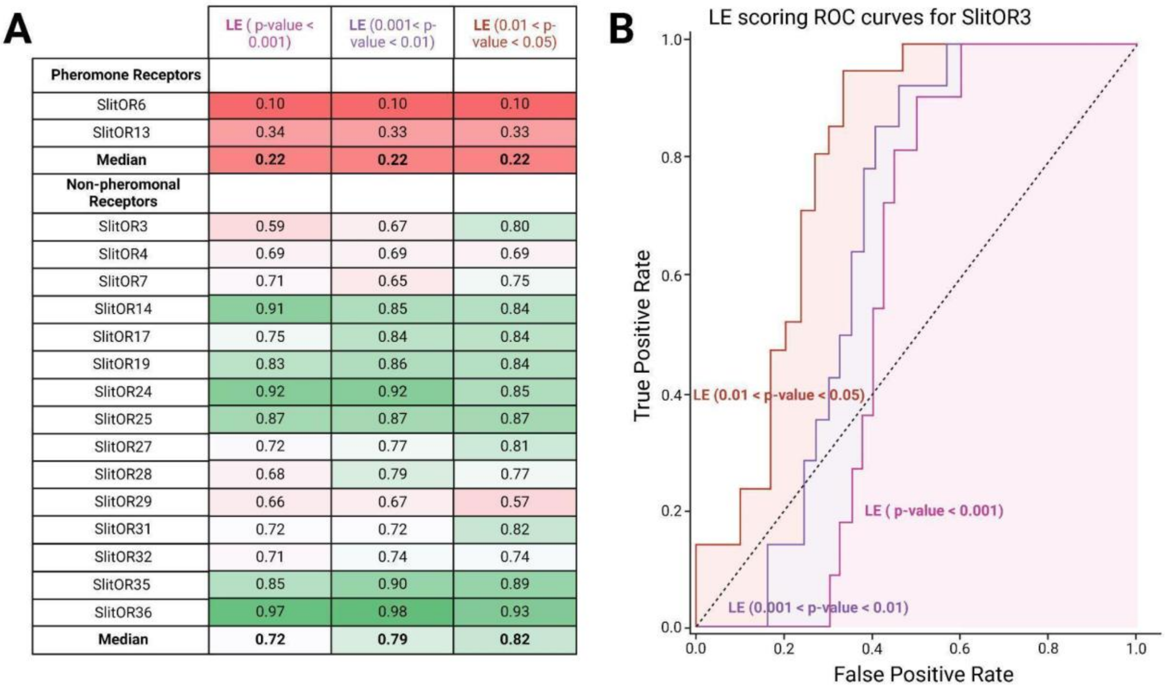

Figure S5: Effect of the p-value threshold on the performance of the *in silico* prediction. (**A**) The table summarizes AUC values from ROC curves using LE scoring function based on optimal poses of 51 molecules across 17 receptors from de Fouchier *et al.*, 2017[20]. Three different thresholds of p-values were considered to determine the activity of the molecule on the SlitORs from experimental data: p-value < 0.001; 0.001 < p-value < 0.01; and 0.01 < p-value < 0.05. The gradient color scheme highlights AUC values, with green indicating higher performance while red are lower ones. (**B**) ROC curves for SlitOR3 show performance with LE scoring function and a p-value < 0.001 (pink); 0.001 < p-value < 0.01 (purple); and 0.01 < p-value < 0.05 (orange).

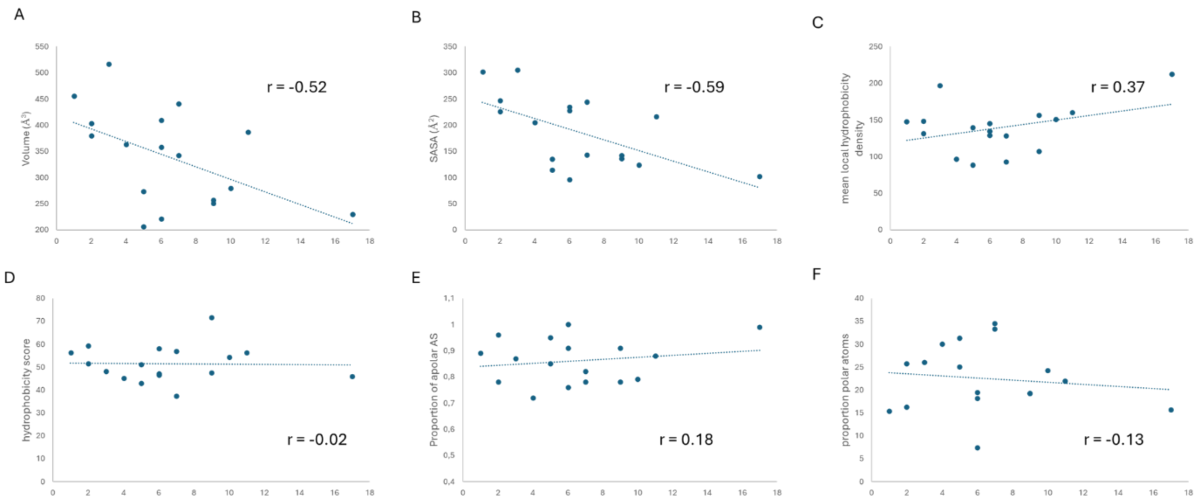

Figure S6: Correlation between pocket descriptors and OR tuning. We have retained the descriptors of the binding cavity that are most relevant from a statistical standpoint (those with the best correlation to the number of ligands per OR) and for their ability to facilitate the deduction of structure-function relationships. The 6 descriptors retained for analysis are as follows: (**A**) Pocket volume, (**B**) Total surface area, (**C**) Mean local hydrophobic density, (**D**) Hydrophobicity score, (**E**) Proportion of apolar alpha spheres and (**F**) Proportion of polar atoms. In the analysis, we did not retain the Volume score, Charge score and Polarity Score as, based on the author comments, these descriptors are extremely approximative and should not be overestimated. The descriptors describing the surface area of the binding pocket (pock_pol_asa, pock_apol_asa, ...) were not significant and too dependent on the absolute value of the Total surface area of the pocket. We decided to calculate a relative value, i.e. the ratio between the apolar surface area and the total surface area. This ratio is significantly correlated to other descriptors (proportion of apolar alpha sphere, Mean local hydrophobic density) already used for the analysis and was finally not kept. The descriptors describing the alpha spheres were also not considered in the analysis as they do not provide a satisfactory explanation and are not easily interpretable. Finally, the amino acid composition of the cavity does not provide a satisfactory explanation, probably due to the limited size of the dataset (17 OR).

Table S8: Correlation matrix. Pearson's correlation coefficient between the number of active ligands (nb_ligands) and pocket descriptors calculated with MDpocket. One has to note that SASA and Volume are highly correlated (r >0.9). To avoid bias in the analysis, we decided to reduce to only 2 variables the linear models (mean_Hyd_dens + SASA or Volume).

		nb_ligands	Volume	SASA	AS_apol_prop	mean_Hyd_dens	Hyd_scor	prop_polar_atm
**Volume**	*Pearson's r*	-0.516	—					
	p-value	0.034	—					
**SASA**	*Pearson's r*	-0.591	0.941	—				
	p-value	0.013	< .001	—				
**AS_apol_prop**	*Pearson's r*	0.180	-0.345	-0.280	—			
	p-value	0.490	0.176	0.276	—			
**mean_Hyd_dens**	*Pearson's r*	0.367	0.187	0.158	0.368	—		
	p-value	0.147	0.473	0.545	0.146	—		
**Hyd_scor**	*Pearson's r*	-0.021	-0.103	0.029	-0.098	-0.183	—	
	p-value	0.937	0.695	0.911	0.709	0.483	—	
**prop_polar_atm**	*Pearson's r*	-0.125	0.265	0.080	-0.462	-0.371	-0.290	—
	p-value	0.634	0.303	0.760	0.062	0.142	0.259	—

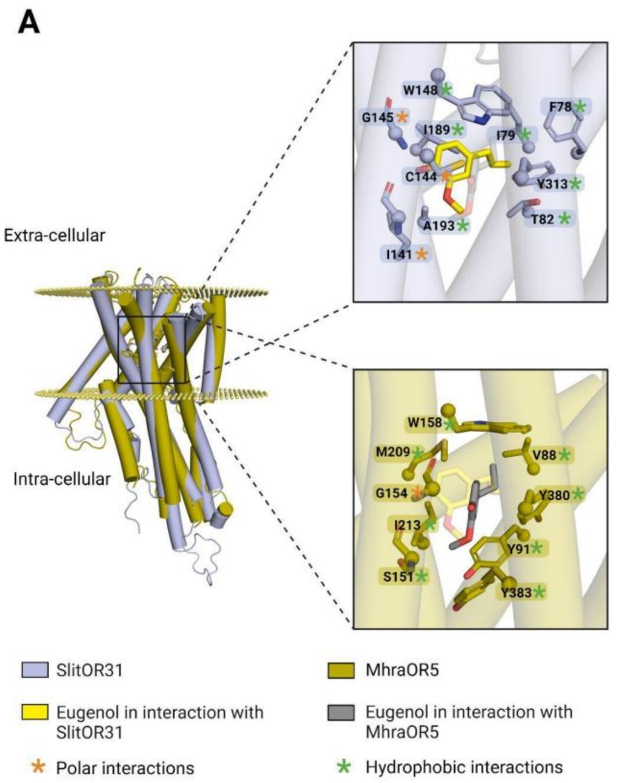

B

SlitOR31	MhraOR5
F78	T87
I79	V88
T82	Y91
I141	S151
C144	G154
G145	W155
W148	W158
I189	M209
A193	I213
Y313	Y380
C316	Y383

Polar interactions Hydrophobic interactions

Figure S7: The recognition of eugenol by the broad receptor MhraOR5 involves fewer polar interactions compared to its recognition by the specific receptor SlitOR31. (**A**) Superposition of the AlphaFold2 model of SlitOR31 (blue) with the cryo-EM structure of MhraOR5 (brown), which binds eugenol, is shown. Orange and green asterisks indicate polar and hydrophobic interactions, respectively, formed between the ligand and residues within the receptor's binding pocket. The backbones of interacting residues are displayed, while non-interacting residues are shown only with their side chains. The receptor's orientation in the membrane was determined using the OPM server[65]. This figure was generated using PyMOL[49]. (**B**) Alignment of the eugenol-binding residues of SlitOR31 and MhraOR5 is presented. The same color code as in (**A**) is used, with hydrophobic interactions shown in green and polar interactions shown in orange.

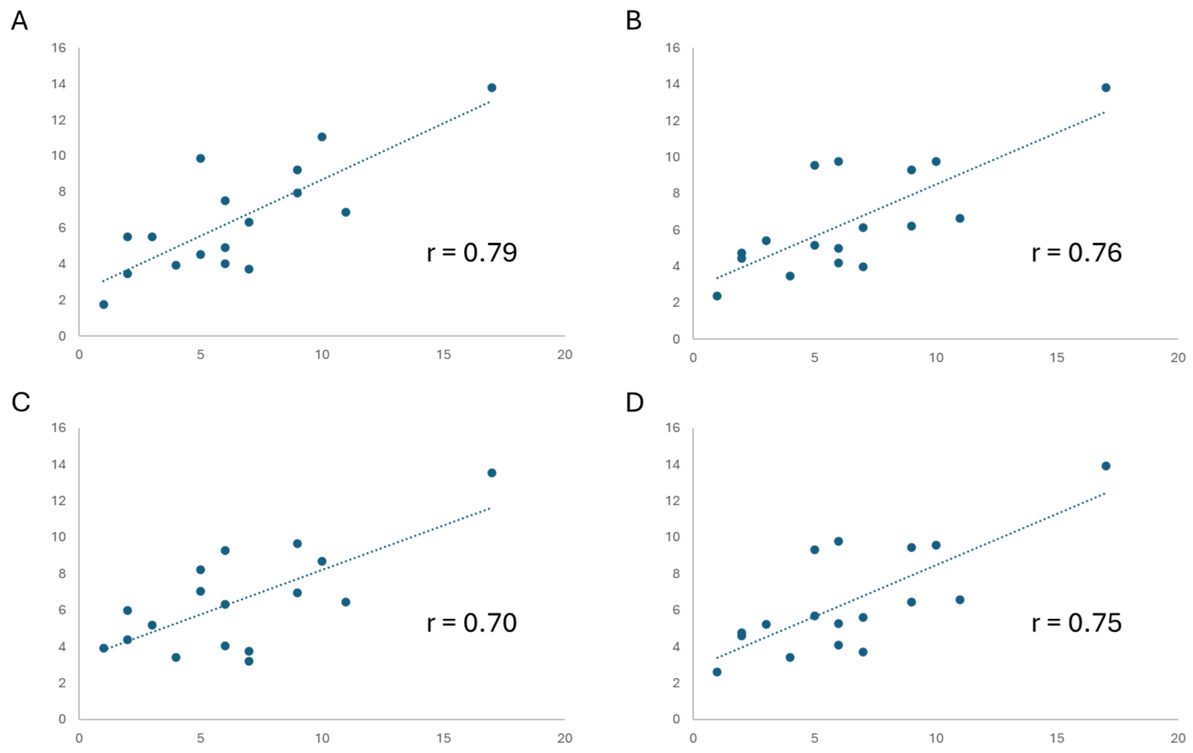

Figure S8: Linear regression models for the prediction of OR broadness based on the description of the binding pocket. The scatter plot represents the predicted *vs* experimental predicted number of ligands per OR for each model. **(A)** The prediction is based on a multiple linear regression (MLR) model including the 6 normalized descriptors as described in the Supplementary Figure 5. (**B**) MLR includes only 3 descriptors, the most significant: Pocket volume, Total surface area and Mean local hydrophobic density. (**C**) MLR includes only 2 descriptors: Volume and Mean local hydrophobic density and (**D**) same as C with Total surface area and Mean local hydrophobic density.

The equation of the model in D) is $NL = -8.07 * SASA + 6.98 * Hyd\_dens + 7.18$ where *NL* means the predicted number of ligands per OR, *SASA* is the total surface area of the binding pocket and *Hyd_dens* is the mean local hydrophobic density. For this model, Pearson's r=0.75 and RMSE=2.53.

Table S9: Performance of the linear regression models. As expected, the model with the higher number of variables yields the best performance. Since three descriptors are too poorly correlated (See Supp. Table 3) and the pocket SASA is highly correlated to the volume, the model with only 2 descriptors reaches similar performance.

Models	r	r^2	RMSE
6 desc	0.79	0.63	2.35
3 desc	0.76	0.57	2.51
2 desc*	0.70	0.49	2.74
2 desc**	0.75	0.57	2.53

* The 2 descriptors used in the model are Volume and Mean hydrophobicity density
** The 2 descriptors used in the model are SASA and Mean hydrophobicity density
